# Development of a park and green space service classification to support park prescribing in Korea

**DOI:** 10.3389/fpubh.2026.1857595

**Published:** 2026-07-30

**Authors:** Hyo-Ju Kim, Daum Kim, Hae-Joon Jung

**Affiliations:** 1 Department of Landscape Architecture, Kyungpook National University, Daegu, Republic of Korea; 2 Graduate School of Public Health, Seoul National University, Seoul, Republic of Korea; 3 Department of Landscape Architecture, College of Engineering, Keimyung University, Daegu, Republic of Korea

**Keywords:** chronic disease prevention, content validity, Delphi method, health-oriented park planning, nature-based health intervention, park prescription, park and green space, service classification

## Abstract

Park Prescription—the formalized referral of patients to park- and nature-based activities by healthcare providers—has gained increasing attention as a strategy for chronic disease prevention and health promotion. Despite the expansion of such programs, a structured classification of park and green space services remains lacking, limiting the practical applicability of Park Prescribing. This issue is particularly important in Korea, where the institutional foundation for implementing Park Prescribing remains limited. Accordingly, this study aimed to develop a classification that conceptualizes parks and green spaces as prescribable service resources and to examine its content validity. A preliminary item set was derived through a review of major international Park Prescribing programs and Park Assessment and Observation Tools. Two rounds of modified Delphi survey were subsequently conducted with a panel of 20 experts, comprising 10 Park and Green Space experts and 10 Healthcare experts. The finalized classification comprises three Domains—Park and Green Space Characteristics, Park and Green Space Spatial Components, and Park and Green Space Activities—and includes 11 Major Items, 36 Middle-Level Items, and 101 Sub-Items. Key items were identified as Accessibility and User Convenience within the Characteristics domain, Pedestrian Space and Rest and Interaction Space within the Spatial Components domain, and Physical Health Activities and Passive Recreation Activities within the Activities domain. The proposed classification provides a foundational framework for linking park and green space services to Park Prescribing. It may support the future implementation of Park Prescribing and serve as a basis for health-oriented park planning and management.

## 1 Introduction

Rapid urbanization and population aging have contributed to the growing burden of non-communicable diseases (NCDs), which have emerged as a major public health challenge. In 2021, NCDs accounted for approximately 75% of all non-pandemic-related deaths worldwide (1). In addition, NCDs are associated with substantial social and economic burdens, including premature mortality, long-term disability, rising healthcare expenditures, productivity losses, and reduced quality of life (2). As disease burdens continue to increase, there is growing recognition that treatment-oriented approaches alone are insufficient to address contemporary health challenges. Consequently, the importance of preventive approaches that consider both the living environment and community resources influencing health has received increasing attention (3).

Within this context, natural environments and green spaces have attracted increasing attention as important resources for health promotion. Exposure to green spaces has been associated with increased physical activity and reduced obesity rates (4–6), lower risks of hypertension and diabetes (7, 8), and reduced levels of depression, stress, and anxiety (9–12). In addition, exposure to green spaces has been shown to contribute to lower medication prescription rates and reduced healthcare expenditures among individuals with chronic diseases (13).

Based on the accumulated empirical evidence, discussions within the international community and healthcare sector have increasingly focused on utilizing natural environments not merely as places for recreation, but as important resources for disease prevention and public health promotion (14, 15). This trend has gradually been integrated into national strategies addressing health equity, mental health, and long-term disease prevention, contributing to the expansion of Park Prescribing programs that connect healthcare services with nature-based activities. Programs such as Park Rx in the United States, Green Social Prescribing in the United Kingdom, PaRx in Canada, and Green Prescription (GRx) in New Zealand involve healthcare providers prescribing nature- and park-based activities to patients to support prevention, treatment, and recovery.

Research on Park Prescribing has expanded across a range of topics, including its conceptual and historical foundations (16), the scope and evidence base of prescriptions (17, 18), physical, mental, and social health effects (19, 20), and implementation and referral pathways within healthcare systems (21–24). In addition, studies evaluating park environments in terms of accessibility, facilities, safety, maintenance conditions, and patterns of use have continued to be conducted within the fields of landscape architecture, environmental design, and public health (25–27). However, these studies have primarily focused on evaluating environmental characteristics and usage conditions of parks, while the issue of systematically conceptualizing parks and green spaces as prescribable service resources from the perspective of Park Prescribing has received comparatively limited attention.

This research gap is also important for the introduction and implementation of Park Prescribing in Korea. As population aging and the burden of chronic diseases continue to increase, the importance of preventive health policies has grown in Korea. Adults aged 65 years and older accounted for 20.3% of the Korean population in 2025, and this proportion is projected to exceed 40% by 2050 (28). In addition, expenditures associated with chronic diseases accounted for 80.3% of total healthcare expenditures in 2024 (29). Meanwhile, Korea has continuously expanded its urban park infrastructure, with urban park area reaching approximately 12.8 m^2^ per capita in 2024. However, this park infrastructure remains insufficiently integrated with the healthcare system, and an institutional framework for implementing Park Prescribing has yet to be established (30, 31).

Accordingly, clinically meaningful implementation of Park Prescribing requires a systematic classification of park and green space services. Therefore, this study aimed to develop a classification system that reconceptualizes the services provided by parks and green spaces from the perspective of prescribable resources, thereby supporting the practical application of Park Prescribing in Korea.

## 2 Methods

### 2.1 Development of the preliminary item set

#### 2.1.1 Source review and item extraction

International Park Prescribing programs and Park Assessment and Observation Tools were reviewed to identify prescribable resources within parks and green spaces. Relevant materials were collected from academic literature, official program websites, and institutional reports, with additional sources identified through reference tracking. Sources were included if they provided information on park and green space service attributes that could be utilized in Park Prescribing.

To identify the park resources and activity types incorporated into existing programs, Park Prescribing program cases were reviewed. Cases were selected primarily from the United Kingdom, where social prescribing has been widely implemented at the national level, and the United States, where Park Prescribing programs are actively operated. Priority was given to programs linked to national healthcare systems or public institutions and whose operational structures and program information could be clearly identified through official documents or related literature. Accordingly, six programs were reviewed: Park Rx America, Kids in Parks Track Trail, Staying Healthy in Nature Everyday, NHS Forest, Nature Prescription, and Our Natural Health Service.

In parallel, existing Park Assessment and Observation Tools were reviewed to identify the physical characteristics and usage patterns of parks. Although these tools were not originally developed as prescription-oriented classification, they include items for assessing or observing environmental characteristics and usage conditions relevant to parks and were therefore used as a basis for preliminary item development. The reviewed tools included the Community Park Audit Tool (CPAT); (32), the Prescription Trails Assessment Worksheet (33), the System for Observing Physical Activity and Recreation in Natural Areas (SOPARNA); (34), and the System for Observing Play and Recreation in Communities (SOPARC); (35). In addition, the Seoul Neighborhood Park Health Promotion Assessment Tool (36) was reviewed to reflect the Korean park and health-promotion context.

Items identified from a total of 11 programs and tools were extracted and cataloged, after which similar or overlapping concepts were consolidated and refined (Supplementary Table 1).

#### 2.1.2 Item organization

To organize the preliminary item structure, Korean institutional and practice-based sources were reviewed. First, legislation related to urban parks and green spaces was reviewed to identify the types and legal scope of parks and green spaces. Second, items related to park attributes, spatial resources, and use conditions were refined with reference to studies on park evaluation and park components, guidelines on landscape and environmental design, and local government park information materials. Third, activity-related items were developed with reference to studies on exercise prescription, guidelines on physical activity, healing and recreation, and local government park program materials. Based on these sources, the preliminary item structure was reorganised to reflect the Korean institutional and practice context.

The preliminary item structure was designed from a service-based perspective that conceptualizes parks as resources that provide health-related services. This approach understands the health-related functions of parks as a set of service attributes arising from the interaction of environmental characteristics, spatial and facility resources, and opportunities for activities that users can undertake (37, 38). Based on this understanding, park and green space services were organized into three Domains: Park and Green Space Characteristics, Park and Green Space Spatial Components, and Park and Green Space Activities. Characteristics refers to conditions related to the suitability of parks for prescription, Spatial Components refers to the types of spaces in which activities take place, and Activities refers to the types of prescribable activities that can be undertaken in parks.

The conceptual relationships and hierarchical structure among items were subsequently reviewed, resulting in a preliminary item structure comprising 14 Major Items, 43 Middle-Level Items, and 135 Sub-Items (Supplementary Table 2). Definitions were prepared to clarify the scope and meaning of the organized items. The preliminary item structure and definitions were then reviewed and refined by two academic experts in landscape architecture and public health. Before Round 1, the draft questionnaire was pilot-tested with three practitioners outside the expert panel to check whether the item definitions, survey instructions, and response format were clearly presented.

### 2.2 Delphi survey

#### 2.2.1 Study design and expert panel

A modified Delphi method was adopted to develop and validate the Park and Green Space Service Classification by achieving expert consensus in an area where empirical criteria remain insufficiently established. The Delphi technique is a structured method for obtaining expert consensus through iterative rounds of evaluation and feedback (39, 40). Unlike the traditional Delphi approach, the modified Delphi method begins with a pre-structured set of items derived from literature reviews, existing materials, and expert input (41). The Delphi technique has also been widely applied in healthcare-related research to establish expert consensus on complex issues (42, 43). Accordingly, this study conducted two survey rounds using a preliminary item structure developed in advance to collect expert judgements and refine the classification.

Panel size and expertise are important factors in ensuring the reliability and validity of Delphi studies. Previous research suggests that a panel of 10 to 18 experts is recommended to achieve meaningful consensus (44), and panel members should possess sufficient expertise relevant to the research topic (45). Based on these recommendations, a panel of 20 experts was assembled, comprising 10 experts from the Healthcare field and 10 experts from the Park and Green Space field. This composition was intended to reflect the interdisciplinary nature of Park Prescribing and to ensure balanced representation of perspectives from both fields.

The expert panel was recruited using purposive sampling. Eligibility criteria required participants to hold at least a master's degree in a relevant field or to have a minimum of 5 years of professional or research experience. Initial recruitment was conducted through professional networks and academic institutions, and additional participants were identified through peer referral when necessary. Healthcare panel comprised experts in preventive medicine, public health, health policy, internal medicine, and pathology, with relevant research or professional experience in public healthcare, disease prevention, and community health. Park and Green Space panel consisted primarily of experts in landscape architecture with research or professional experience in the planning, design, and management of parks, green spaces, and landscapes. The general characteristics of the expert panel are presented in Table 1.

**Table 1:** General characteristics of the expert panel.

Section		Frequency (*n*)	Percentage (%)
Area of expertise	Healthcare	10	50.0
	Park and green space	10	50.0
Age	30–39 years	5	25.0
	40–49 years	10	50.0
	50–59 years	4	20.0
	60 years or older	1	5.0
Educational attainment	Doctoral degree	13	65.0
	Master's degree	7	35.0
Institution type	Universities	10	50.0
	Hospitals and clinics	4	20.0
	Research institutes	3	15.0
	Private sectors	2	10.0
	Public institutions	1	5.0
Work experience	Less than 5 years	3	15.0
	5–9 years	4	20.0
	10–19 years	8	40.0
	20 years or more	5	25.0

All prospective participants were provided with written information describing the study objectives and procedures before confirming their participation. Participation was voluntary, and all panel members provided informed consent prior to the survey. Anonymity and confidentiality were maintained throughout the study, and individual identities and responses were kept confidential. For the purpose of expert group-specific analysis, only participants' disciplinary affiliation (Healthcare or Park and Green Space) was separately recorded by the research team. Ethical approval for the study was obtained from Keimyung University (No. 40525-202201-HR-095-01).

#### 2.2.2 Survey procedure

**Figure 1:**
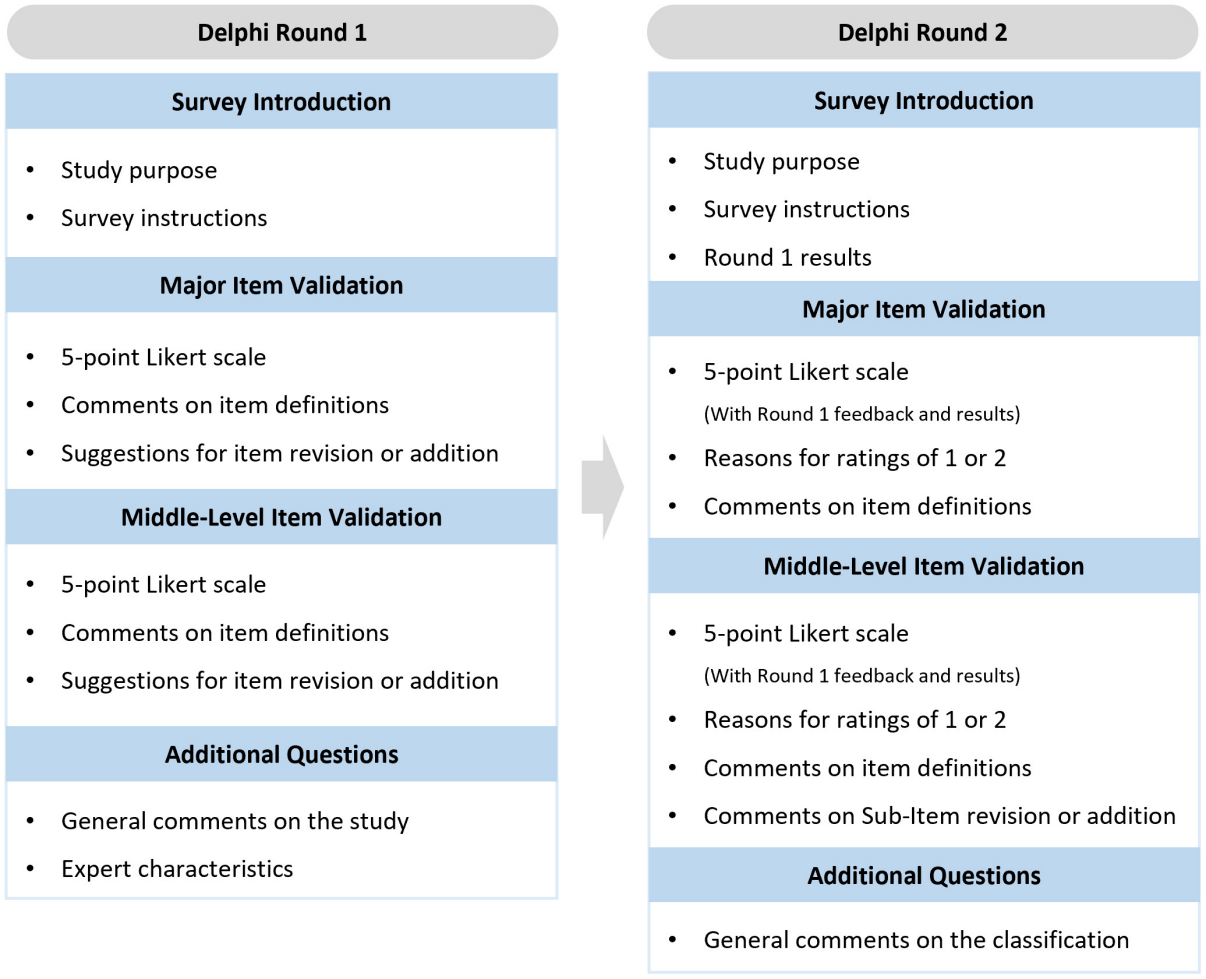
Structure of the Delphi survey.

The two-round Delphi survey procedure is illustrated in Figure 1. Both rounds were conducted online, and survey links were distributed to the expert panel via email. Round 2 was conducted approximately 6 weeks after completion of Round 1. All 20 panel members completed both survey rounds.

Round 1 was conducted to assess the preliminary validity of the initial item structure. During the survey introduction stage, participants were provided with the study purpose and survey instructions. In the Major Item and Middle-Level Item validation stages, conceptual definitions of each item were presented, and experts were asked to evaluate their importance using a five-point Likert scale. Participants were also invited to provide comments on item definitions and suggestions for item revision or addition. Sub-Items were not independently evaluated but were presented as illustrative examples to clarify the scope and meaning of each Middle-Level Item. The survey concluded with questions regarding general comments on the study and expert characteristics.

Round 2 was conducted to re-evaluate the validity of the revised item structure following incorporation of the Round 1 results and expert feedback. During the survey introduction stage, participants were provided with the study purpose, survey instructions, and a summary of the Round 1 results. In the Major Item and Middle-Level Item validation stages, conceptual definitions were again presented and evaluated using the same five-point Likert scale. To facilitate informed re-evaluation, participants were provided with a summary of item revisions made after the Round 1, group-level statistics from the Round 1 (mean, standard deviation, median, and interquartile range), and their own previous responses. Participants who assigned a low rating of 1 or 2 were asked to provide reasons for their ratings. Comments on item definitions were also collected. Furthermore, comments regarding the revision or addition of Sub-Items were collected during the Middle-Level Item validation stage. This procedure was not included in the Round 1 survey and was intended to refine the illustrative examples and scope of the final classification. The survey concluded with an opportunity for participants to provide general comments on the overall classification.

#### 2.2.3 Data analysis and item revision

The Delphi survey data were analyzed through two procedures: quantitative evaluation and item revision.

For the quantitative evaluation, mean scores, standard deviations, and CVRs (Content Validity Ratios) were calculated for each item in both Round 1 and Round 2. The reliability of the survey instrument was assessed using Cronbach's α. Mean scores were used to evaluate the perceived importance of each item, with a threshold of ≥ 3.0. CVR was used to assess content validity among the expert panel, with the minimum acceptable value set at CVR ≥ 0.42 for a panel of 20 experts (46). Although acceptable thresholds for Cronbach's α vary according to the nature of the study (47), values in the range of 0.50–0.60 are generally considered acceptable in exploratory research involving broad and multidimensional constructs (48, 49). In Round 2, convergence, consensus, and stability were additionally calculated to examine changes in expert opinion and the level of agreement. Convergence was used to assess the concentration of expert responses, consensus to evaluate the degree of collective agreement based on the response distribution, and stability to assess the consistency of responses across rounds. Thresholds were established with reference to the methodological literature on Delphi studies (50) and defined as: Convergence ≤ 0.50, Consensus ≥ 0.75, and Stability ≤ 0.50.

The item revision process was conducted as follows. Major Items and Middle-Level Items that did not meet the quantitative criteria described above were not automatically regarded as inappropriate. Instead, they were classified as items requiring further review, considering the possibility that elements potentially relevant to practical implementation contexts may not have been fully captured through the quantitative evaluation alone. Four criteria were considered in the revision process: (a) results of the group-specific analyses, (b) conceptual necessity identified through expert feedback, (c) relationships among items, and (d) evidence from international Park Prescribing programs and their potential applicability within the Korean context. For the group-specific analyses, a CVR threshold of ≥0.62 was applied because each expert group consisted of 10 participants (46). Based on these considerations, decisions were made regarding the deletion, addition, integration, subdivision, and relocation of Major Items and Middle-Level Items. Items that did not meet the quantitative criteria but were ultimately retained in the final classification were designated as provisionally retained items. The names and definitions of Major Items and Middle-Level Items were also revised when expert feedback indicated conceptual ambiguity or the need for terminological refinement. Sub-Items were not subjected to separate quantitative validity assessment in the Delphi process. Instead, Sub-Items were reviewed in terms of their functional appropriateness within the corresponding Middle-Level Item, and their names and contents were revised based on expert feedback.

## 3 Results

### 3.1 Major item results and revisions

#### 3.1.1 Round 1

Evaluation of the Major Items in Round 1 showed that 10 of the 14 Major Items met the predefined acceptance criteria for both mean score and CVR (Table 2). Within the Characteristics domain, all items met the criteria. In contrast, [Exercise Space], [Water Related Space], and [Cultural Space] within the Spatial Components domain, and [Community Program Activities] within the Activities domain, failed to meet the CVR criterion. Cronbach's α was 0.570 for the Characteristics domain, 0.506 for the Spatial Components domain, and 0.742 for the Activities domain.

**Table 2:** Round 1 Delphi survey results for major items.

Domain	Major item	Mean	SD	CVR	Cronbach's α
Park and green space characteristics	General characteristics	4.050	0.740	0.500	0.570
	Accessibility	4.800	0.400	1.000	
	Safety	4.300	0.557	0.900	
	Usability	4.250	0.698	0.700	
	Program	3.850	0.853	0.500	
	Naturalness	4.150	0.853	0.600	
Park and green space spatial components	Pedestrian space	4.850	0.357	1.000	0.506
	Exercise space	4.050	0.805	0.400	
	Rest space	4.650	0.572	0.900	
	Water related space	3.500	0.975	0.000	
	Cultural space	3.600	0.860	−0.100	
Park and green space activities	Physical and mental health activities	4.550	0.805	0.800	0.742
	Nature based activities	4.450	0.740	0.700	
	Community program activities	3.950	0.805	0.300	

Shaded cells indicate items that did not meet the predefined criteria. The criteria were Mean ≥ 3.0 and CVR ≥ 0.42.

**Figure 2:**
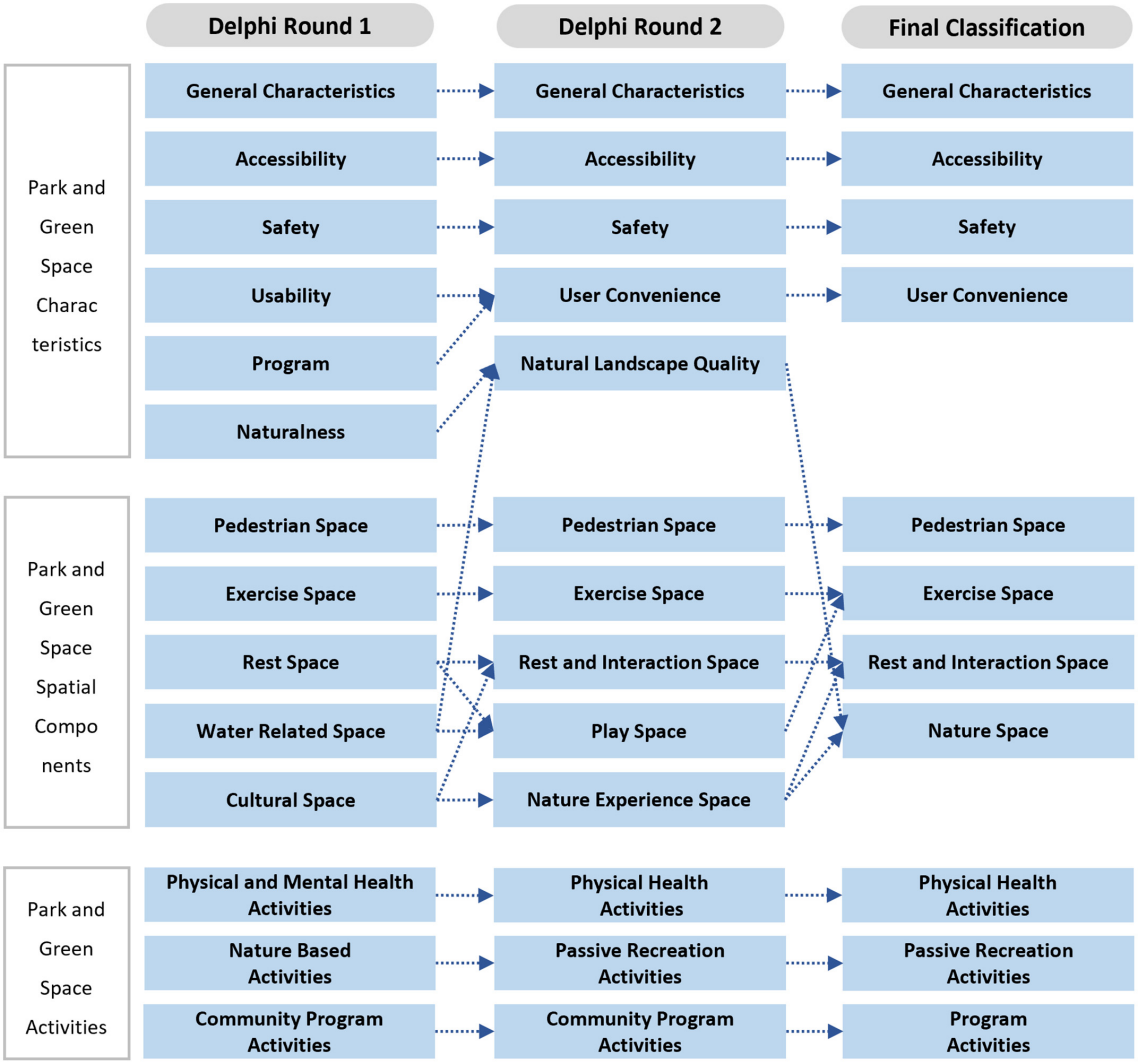
Refinement of major items across Delphi rounds.

Following Round 1, the Major Item structure was revised based on the quantitative results and expert feedback (Figure 2). The major revisions were as follows.

Within the Characteristics domain, [Program] was integrated with [Usability] and restructured as [User Convenience], reflecting expert feedback that programs function more as operational elements supporting park use than as an independent Major Item. [Naturalness] was renamed [Natural Landscape Quality] to provide a more specific and interpretable concept.

Within the Spatial Components domain, [Rest Space] was revised to [Rest and Interaction Space] to better reflect the social functions of green spaces. [Water Related Space] was removed in response to expert feedback indicating its limited relevance to Park Prescribing, whereas [Exercise Space] was retained for further review. [Play Space] and [Nature Experience Space] were added to more explicitly represent opportunities for play and nature-based experiences. [Cultural Space] was reclassified as a Middle-Level Item in to better reflect its role as a specific spatial component.

Within the Activities domain, the activity structure was reorganized based on expert feedback suggesting that park and green space activities should be classified according to their relevance to physical activity promotion, psychological rest and restoration, and program-based participation. Accordingly, [Physical and Mental Health Activities] was renamed [Physical Health Activities], and [Nature Based Activities] was renamed [Passive Recreation Activities]. [Community Program Activities] was retained for further review.

#### 3.1.2 Round 2

Evaluation of the Major Items in Round 2 showed that 10 of the 13 Major Items met the predefined acceptance criteria for mean score, CVR, convergence, consensus, and stability (Table 3). Within the Characteristics domain, all items met the criteria. In contrast, within the Spatial Components domain, [Play Space] failed to meet the CVR criterion, while [Nature Experience Space] failed to meet the convergence and consensus criteria. Within the Activities domain, [Community Program Activities] failed to meet the CVR criterion. Cronbach's α was 0.670 for the Characteristics domain, 0.631 for the Spatial Components domain, and 0.748 for the Activities domain.

**Table 3:** Round 2 Delphi survey results for major items.

Domain	Major item	Mean	SD	CVR	Convergence	Consensus	Stability	Cronbach's α
Park and green space characteristics	General characteristics	4.200	0.678	0.700	0.500	0.750	0.161	0.670
	Accessibility	4.750	0.433	1.000	0.125	0.950	0.091	
	Safety	4.350	0.654	0.800	0.500	0.750	0.150	
	User convenience	4.350	0.572	0.900	0.500	0.750	0.131	
	Natural landscape quality	4.250	0.766	0.600	0.500	0.750	0.180	
Park and green space spatial components	Pedestrian space	4.800	0.400	1.000	0.000	1.000	0.083	0.631
	Exercise space	4.150	0.572	0.800	0.125	0.938	0.138	
	Rest and interaction space	4.500	0.500	1.000	0.500	0.778	0.111	
	Play space	3.700	0.458	0.400				
	Nature experience space	4.000	0.837	0.500	0.625	0.688	0.209	
Park and green space activities	Physical health activities	4.550	0.589	0.900	0.500	0.800	0.129	0.748
	Passive recreation activities	4.300	0.640	0.800	0.500	0.750	0.149	
	Community program activities	3.800	0.678	0.300				

Shaded cells indicate items that did not meet the predefined criteria. The criteria were Mean ≥ 3.0, CVR ≥ 0.42, Convergence ≤ 0.50, Consensus ≥ 0.75, and Stability ≤ 0.50. Convergence, consensus, and stability were not evaluated for items with CVR < 0.42.

Following Round 2, the Major Item structure was finalized based on the quantitative results and expert feedback (Figure 2). During this process, items that failed to meet the acceptance criteria were re-examined according to the four item revision criteria described in the Methods section, and selected items were classified as provisionally retained items. The major revisions were as follows.

Within the Spatial Components domain, [Natural Landscape Quality] and [Nature Experience Space] were consolidated into [Nature Space] to reduce conceptual overlap among nature-related items and to more comprehensively represent both natural characteristics and nature-based spaces. In addition, [Play Space] was reclassified as a Middle-Level Item in response to expert feedback indicating that its exercise-related functions could overlap with those of [Exercise Space].

Within the Activities domain, [Community Program Activities] did not meet the acceptance criteria. However, the related Middle-Level Item, [Program Offerings], met the criteria, and program-based activities were identified as a key implementation component in international Park Prescribing programs. Accordingly, the item was retained and subsequently renamed [Program Activities].

### 3.2 Middle-level item results and revisions

#### 3.2.1 Round 1

Evaluation of the Middle-Level Items in Round 1 showed that 22 of the 43 Middle-Level Items met the predefined acceptance criteria for both mean score and CVR (Table 4). The Characteristics domain showed the highest proportion of items meeting the criteria, whereas the Spatial Components and Activities domains contained a relatively larger number of items that failed to meet the criteria. Cronbach's α was 0.717 for the Characteristics domain, 0.624 for the Spatial Components domain, and 0.846 for the Activities domain.

**Table 4:** Round 1 Delphi survey results for middle-level items.

Domain	Major item	Middle-level item	Mean	SD	CVR	Cronbach's α
Park and green space characteristics	General characteristics	Park location	4.250	1.043	0.600	0.717
		Park type	3.750	0.887	0.100	
		Park area	3.750	0.622	0.500	
	Accessibility	Access distance	4.550	0.805	0.600	
		Public transportation	4.400	0.583	0.900	
		parking lots	4.050	0.740	0.500	
	Safety	Lighting facilities	4.100	0.831	0.600	
		Management facilities	4.200	0.678	0.700	
	Usability	Service facilities	4.150	0.654	0.700	
		Pet facilities	3.600	0.970	0.200	
		Convenience facilities	4.400	0.583	0.900	
	Program	Personal program	3.950	0.669	0.500	
		Community program	4.050	0.740	0.700	
	Naturalness	Natural landscape	4.250	0.698	0.700	
		Green space	4.300	0.640	0.800	
		Green coverage ratio	3.800	0.748	0.400	
Park and green space spatial components	Pedestrian space	Difficulty level	4.250	0.766	0.800	0.624
		Pavement type	3.900	0.700	0.400	
		Length	3.900	0.624	0.500	
		Width	3.850	0.963	0.100	
		Trail layout	3.100	0.768	−0.500	
		Activity categories	4.300	0.557	0.900	
	Exercise space	Fitness facilities	4.050	0.865	0.300	
		Sports facilities	3.950	0.921	0.300	
		Open space	4.300	0.714	0.700	
	Rest space	Rest facilities	4.500	0.742	0.700	
		Play facilities	3.850	0.853	0.100	
	Water related space	Water features	3.600	1.068	0.200	
		Waterfront space	3.650	1.014	0.200	
	Cultural space	Cultural facilities	3.100	0.943	−0.400	
		Leisure facilities	3.500	1.025	0.100	
		Community facilities	4.100	0.943	0.400	
Park and green space activities	Physical and mental health activities	Aerobic exercise	4.700	.458	1.000	0.846
		Resistance exercise	3.850	1.108	0.300	
		Flexibility exercise	3.850	1.014	0.300	
		Sports activities	3.600	1.114	0.100	
		Rest and recreational activities	4.250	0.766	0.600	
	Nature based activities	Landscape appreciation activities	4.150	0.654	0.700	
		Nature observation activities	3.950	0.865	0.400	
		Nature learning activities	4.250	0.698	0.700	
	Community program activities	Cultural activities	3.400	0.860	0.000	
		Educational activities	3.400	0.583	−0.100	
		Volunteer activities	3.600	0.735	0.100	

Shaded cells indicate items that did not meet the predefined criteria. The criteria were Mean ≥ 3.0 and CVR ≥0.42.

Following Round 1, the Middle-Level Item structure was revised based on the quantitative results and expert feedback (Supplementary Table 3). The major revisions were as follows.

First, several items were revised to improve conceptual clarity and terminological precision. [Access Distance] was renamed [Pedestrian Accessibility] in response to expert feedback that actual user accessibility should be considered rather than relying solely on legal standards. Experts also noted that [Lighting Facilities] and [Management Facilities] alone were insufficient to represent the conditions necessary for safe park use. Accordingly, these items were revised as [Safe Use Conditions] and [Monitoring and Emergency Support], respectively. In addition, [Convenience Facilities] was renamed [Vulnerable Group Facilities] to more explicitly reflect accessibility for vulnerable populations. [Community Facilities] was also renamed [Cultivation Facilities], as experts considered it to be more closely associated with urban agriculture and cultivation activities than with general community facilities.

Second, item integration was undertaken to address conceptual overlap and unclear hierarchical relationships among items. [Park Location] and [Park Type] were merged into [Type] following expert feedback that the distinction between the two was ambiguous. [Personal Program] and [Community Program] were consolidated into [Program Offerings], to better reflect program availability. [Natural Landscape] and [Green Space] were reorganized as [Green and Blue Space Type] to provide a more direct representation of natural environment types and to resolve ambiguity in their conceptual hierarchy.

Third, item subdivision was performed to better reflect differences in intended use and functional characteristics. Because [Open Space] encompassed both exercise and rest functions, it was divided into [Open Exercise Areas] and [Open Rest Areas]. [Nature Learning Activities] was subdivided into [Nature Observation Activities] and [Nature Experience Activities] to distinguish general observation-based activities from more specific educational or interpretive experiences.

Fourth, several new items were added to improve the applicability of the classification to Park Prescribing. These included [Crowding Level] to assess the degree of user congestion, [Cost Accessibility] to reflect the economic burden associated with park use, and [Risk Prevention] to capture functions related to risk communication and accident prevention. In addition, [Landscape Characteristics], [Ecological Quality], and [Observation Facilities] were added to more explicitly represent landscape attributes, ecological health, and opportunities for nature observation.

Finally, item deletion focused on items that failed to meet the acceptance criteria or were considered insufficiently reflective of the intended concept. [Green Coverage Ratio] was removed following expert feedback that the amount of shade or vegetation cover does not necessarily reflect the level of naturalness. [Width] and [Trail Layout] were also removed because they failed to meet the acceptance criteria.

#### 3.2.2 Round 2

Evaluation of the Middle-Level Items in Round 2 showed that 25 of the 40 Middle-Level Items met the predefined acceptance criteria for mean score, CVR, convergence, consensus, and stability (Table 5). In contrast, Middle-Level Items that failed to meet the criteria were mainly concentrated under [Play Space] and [Nature Experience Space] within the Spatial Components domain and under [Community Program Activities] within the Activities domain. Cronbach's α was 0.719 for the Characteristics domain, 0.877 for the Spatial Components domain, and 0.687 for the Activities domain.

**Table 5:** Round 2 Delphi survey results for middle-level items.

Domain	Major item	Middle-level item	Mean	SD	CVR	Convergence	Consensus	Stability	Cronbach's α
Park and green space characteristics	General characteristics	Type	4.200	0.812	0.700	0.500	0.750	0.193	0.719
		Area	3.750	0.536	0.400				
		Crowding level	3.950	0.589	0.600	0.000	1.000	0.149	
	Accessibility	Pedestrian accessibility	4.700	0.557	0.900	0.125	0.950	0.119	
		Public transportation accessibility	4.350	0.572	0.900	0.500	0.750	0.131	
		Vehicle accessibility	4.150	0.572	0.800	0.125	0.938	0.138	
		Cost accessibility	4.350	0.572	0.900	0.500	0.750	0.131	
	Safety	Monitoring and emergency support	4.250	0.622	0.800	0.500	0.750	0.146	
		Risk prevention	4.200	0.510	0.900	0.125	0.938	0.121	
		Safe use conditions	4.450	0.589	0.900	0.500	0.778	0.132	
	User convenience	Amenities	4.150	0.572	0.800	0.125	0.938	0.138	
		Vulnerable group facilities	4.550	0.497	1.000	0.500	0.800	0.109	
		Program offerings	4.000	0.632	0.600	0.000	1.000	0.158	
	Natural landscape quality	Green and blue space type	4.200	0.748	0.600	0.500	0.750	0.178	
		Landscape characteristics	4.000	0.707	0.700	0.000	1.000	0.177	
		Ecological quality	3.800	0.748	0.400				
Park and green space spatial components	Pedestrian space	Difficulty level	4.250	0.433	1.000	0.125	0.938	0.102	0.877
		Length	4.000	0.548	0.700	0.000	1.000	0.137	
		Activity categories	4.300	0.458	1.000	0.500	0.750	0.107	
	Exercise space	Fitness facilities	4.100	0.768	0.500	0.625	0.688	0.187	
		Sports facilities	3.850	0.726	0.300				
		Open exercise areas	4.350	0.654	0.800	0.500	0.750	0.150	
	Rest and interaction space	Rest facilities	4.400	0.663	0.800	0.500	0.778	0.151	
		Open rest areas	4.000	0.707	0.500	0.250	0.875	0.177	
		Cultural facilities	3.750	0.622	0.300				
	Play space	Age specific play facilities	3.850	0.654	0.400				
		Seasonal play facilities	3.300	0.843	−0.200				
		Nature play facilities	3.850	0.726	0.300				
	Nature experience space	Observation facilities	3.700	0.714	0.100				
		Leisure facilities	3.750	0.536	0.400				
		Cultivation facilities	3.650	0.654	0.100				
Park and green space activities	Physical health activities	Aerobic exercise	4.500	0.671	0.800	0.500	0.800	0.149	0.687
		Strength training	4.100	0.831	0.600	0.500	0.750	0.203	
		Sports and group exercise	3.900	0.768	.500	0.125	0.938	0.197	
	Passive recreation activities	Landscape appreciation activities	4.250	0.622	0.800	0.500	0.750	0.146	
		Nature observation activities	3.950	0.740	0.400				
		Rest and recreational activities	4.050	0.669	0.600	0.125	0.938	0.165	
	Community program activities	Educational and cultural activities	3.650	0.654	0.300				
		Nature experience activities	3.800	0.678	0.300				
		Volunteer activities	3.700	0.714	0.100				

Shaded cells indicate items that did not meet the predefined criteria. The criteria were Mean ≥ 3.0, CVR ≥ 0.42, Convergence ≤ 0.50, Consensus ≥ 0.75, and Stability ≤ 0.50. Convergence, consensus, and stability were not evaluated for items with CVR < 0.42.

Following Round 2, the Middle-Level Item structure was finalized based on the quantitative results and expert feedback (Supplementary Table 3). During this process, items that failed to meet the acceptance criteria were re-examined according to the four item revision criteria described in the Methods section, and selected items were classified as provisionally retained items. The major revisions were as follows.

Within the Characteristics domain, [Area] was retained despite narrowly failing to meet the acceptance criteria in Round 2, as it had met the criteria in Round 1 and was considered a fundamental attribute for describing the size characteristics of parks and green spaces. In contrast, [Ecological Quality] was removed based on expert feedback indicating that it is more applicable to natural parks than to general urban parks, is difficult to assess and verify at the individual park level, and is of relatively secondary importance in the context of Park Prescribing. In addition, [Crowding Level] was renamed [User Density], and [Landscape Characteristics] was renamed [Natural Landscape Type] to improve conceptual clarity.

Within the Spatial Components domain, [Fitness Facilities] was retained because it met the CVR criterion within the Healthcare group and was considered functionally related to [Aerobic Exercise] and [Strength Training]. [Sports Facilities] was also retained because it provides the spatial infrastructure necessary for [Sports and Group Exercise]. [Age Specific Play Facilities], [Seasonal Play Facilities], and [Nature Play Facilities] received relatively high evaluations from the Park and Green Space group and were integrated into [Play Facilities] because they shared the common function of supporting play activities. [Leisure Facilities] was retained because it met the CVR criterion within the Park and Green Space group and demonstrated a high level of consensus. [Observation Facilities] and [Cultivation Facilities] were also retained in consideration of their use not only in international Park Prescribing programs but also in nature-based therapeutic practices in Korea. To reduce overlap and improve the placement of items within the classification, [Observation Facilities] was redefined as [Nature Experience Facilities] while [Cultivation Facilities] was redefined as [Gardening Facilities].

Within the Activities domain, [Nature Observation Activities] was retained because it received relatively high evaluations from the Park and Green Space group and is actively implemented in park-based programs in Korea. [Educational and Cultural Activities], [Nature Experience Activities], and [Volunteer Activities] were retained because of their conceptual relationship with [Program Offerings]. In addition, [Educational and Cultural Activities] was renamed [Physical and Cultural Activities], [Program Offerings] was renamed [Program Availability], and [Activity Categories] was renamed [Activity Type] to provide clearer and more interpretable concepts.

### 3.3 Final park and green space service classification

Following two rounds of survey and refinement, the preliminary item structure was refined from 14 Major Items, 43 Middle-Level Items, and 135 Sub-Items to a final structure comprising 11 Major Items, 36 Middle-Level Items, and 101 Sub-Items.

The final Park and Green Space Service Classification is organized into three domains (Table 6). The Characteristics domain comprises four Major Items: [General Characteristics], [Accessibility], [Safety], and [User Convenience]. The Spatial Components domain comprises four Major Items: [Pedestrian Space], [Exercise Space], [Rest and Interaction Space], and [Nature Space]. The Activities domain comprises three Major Items: [Physical Health Activities], [Passive Recreation Activities], and [Program Activities].

**Table 6:** Final park and green space service classification.

Domain	Major item	Middle-level item
Park and green space characteristics	General characteristics	Type
		Area
		User density
	Accessibility	Pedestrian accessibility
		Public transportation accessibility
		Vehicle accessibility
		Cost accessibility
	Safety	Monitoring and emergency support
		Risk prevention
		Safe use conditions
	User convenience	Amenities
		Vulnerable group facilities
		Program availability
Park and green space spatial components	Pedestrian space	Difficulty level
		Length
		Activity type
	Exercise space	Fitness facilities
		Sports facilities
		Open exercise areas
		Play facilities
	Rest and interaction space	Rest facilities
		Open rest areas
		Leisure facilities
		Gardening facilities
	Nature space	Green and blue space type
		Natural landscape type
		Nature experience facilities
Park and green space activities	Physical health activities	Aerobic exercise
		Strength training
		Sports and group exercise
	Passive recreation activities	Landscape appreciation activities
		Nature observation activities
		Rest and recreational activities
	Program activities	Physical and cultural activities
		Nature experience activities
		Volunteer activities

Shaded cells indicate provisionally retained major or middle-level items.

Items that did not meet the predefined criteria but were retained following additional review were classified as provisionally retained items. Definitions of each Major Item and Middle-Level Item, together with the complete list of 101 Sub-Items, are presented in Supplementary Table 4.

## 4 Discussion

### 4.1 Core components for park prescribing

Several core components for Park Prescribing received consistent support across the survey rounds. These included [Accessibility] and [User Convenience] within the Characteristics domain, [Pedestrian Space] and [Rest and Interaction Space] within the Spatial Components domain, and, within the Activities domain, [Physical Health Activities], particularly [Aerobic Exercise], and [Passive Recreation Activities], particularly [Landscape Appreciation Activities]. The results suggest that the experts regarded routine physical activity, such as walking, and restorative experiences derived from contact with natural environments as core components of Park Prescribing. This interpretation is consistent with previous studies reporting that engagement with natural environments may contribute to health through both physical activity and psychological restoration (9, 51).

Comments from the expert panel further indicated that the primary value of parks and green spaces was considered to lie in the opportunities they provide for rest, relaxation, and emotional well-being through contact with nature, whereas facilities and programs were viewed as complementary elements that support these experiences. Accordingly, the present classification may be understood as a framework that places accessibility in everyday life, routine physical activity, and nature-based restorative experiences at the core of Park Prescribing, while also incorporating facility- and program-related components as supporting elements.

Expert comments also suggested that the key resources for Park Prescribing should not necessarily be restricted to spaces formally designated as parks, but rather defined according to their actual usability and relevance to prescription objectives. For example, some experts noted that urban green spaces that perform park-like functions, such as publicly accessible open spaces and rooftop gardens, could be considered prescribable resources even when they are not institutionally classified as parks. This suggests that the value of Park Prescribing lies less in a specific type of space than in providing opportunities for contact with natural environments that can contribute to health promotion.

### 4.2 Components requiring additional consideration

Across the two rounds of the survey, items that failed to meet the acceptance criteria were distributed across several domains in Round 1 but became concentrated within a limited number of item groups in Round 2. Notably, many of the items that failed to meet the criteria in Round 2 had been newly introduced or revised based on expert feedback received in Round 1. This suggests that, although the necessity of these items was recognized by some experts, sufficient consensus had not yet been reached regarding their inclusion and scope. It also indicates that newly introduced items may have limited opportunities to achieve consensus within a Delphi process involving only a small number of iterative review rounds. Therefore, items that failed to meet the criteria should not be interpreted simply as candidates for exclusion, but rather as components requiring additional consideration.

The group-specific analysis revealed differences in the evaluation of several items between the expert groups (Supplementary Table 5). [Ecological Quality], [Nature Play Facilities], and [Leisure Facilities] were evaluated more positively by the Park and Green Space group. In contrast, items more directly related to supporting physical activity, such as [Fitness Facilities], received relatively higher evaluations from the Healthcare group. These findings suggest that Park Prescribing represents an inherently interdisciplinary approach that requires collaboration between the Healthcare and Park and Green Space fields (22, 23).

[Program Activities] was also identified as a component requiring additional consideration. Sufficient consensus was not reached regarding whether specific activities delivered through programs should be included as components of park services. Experts noted that program activities, unlike the physical environment or facilities of a park, may vary according to program design and operating organizations, and that some activities can also be conducted outside park settings. However, international Park Prescribing programs indicate that community-based programs can serve as important implementation mechanisms. Some experts also noted that patients may face practical difficulties in maintaining prescribed activities independently. Building on this view, community-based programs may help support continued engagement by providing organized opportunities for patients to carry out prescribed activities. Accordingly, [Program Activities] may be understood as a component that requires further consideration regarding the scope of its inclusion within park services.

In the case of [User Density], the primary issue concerned not the necessity of the item itself, but rather the methods by which it could be measured and communicated. Some experts raised concerns regarding its dynamic nature, as density levels may vary substantially depending on time, season, and other situational factors, making it difficult to provide as a fixed attribute of a park. Nevertheless, [User Density] may act as a source of psychological discomfort or avoidance for some users and therefore warrants consideration when assessing the suitability of parks for prescription purposes. User Density may therefore be interpreted as a potentially important component that could be utilized in conjunction with time-specific visitation data or real-time monitoring systems in future applications.

### 4.3 Distinctive features of the park and green space service classification

The classification developed in this study shares a conceptual foundation with existing Park Assessment and Observation Tools, but extends their scope to support the practical implementation of Park Prescribing. In particular, the differences can be understood in terms of the perspective through which items are organized, the primary users, and the scope of considerations incorporated into the classification.

First, the classification differs in the perspective through which items are organized. Existing tools are designed to assess or observe specific aspects of parks, such as physical environments, facility conditions, or patterns of use, according to their respective objectives. For example, CPAT evaluates facility conditions, accessibility, and park use environments, whereas SOPARC and SOPARNA focus on observing patterns of use, activity types, and levels of physical activity (32, 34, 35). In contrast, the present classification integrates and reorganizes these environmental conditions and activity opportunities into service attributes that can be utilized within Park Prescribing, structured across the domains of Characteristics, Spatial Components, and Activities.

Second, the classification differs in its primary users. Whereas existing tools are primarily used by researchers, park managers, and policymakers to support environmental assessment and park management, the present classification may assist healthcare providers in reviewing and selecting park and green space service attributes that are appropriate for individual patients' health goals and characteristics within the Park Prescribing process.

Third, the classification differs in the scope of considerations it incorporates. Existing tools may include information related to accessibility and user characteristics that influence park use, but they are not specifically designed to interpret such information in terms of users' ability to use prescribed resources or potential barriers to participation within the Park Prescribing process. In contrast, the present classification incorporates items such as [Cost Accessibility], [Vulnerable Group Facilities], and [Program Availability], enabling healthcare providers to consider economic burden, accessibility for vulnerable user groups, and conditions that may support prescription adherence when selecting appropriate park and green space service attributes. Previous studies on Park Prescribing have likewise identified access barriers and adherence-related challenges as important implementation issues (21, 52), suggesting that these considerations should also be reflected in the classification of prescribable park and green space resources.

### 4.4 Clinical and policy implications

The classification may contribute to the development of both the clinical application and policy foundation of Park Prescribing in Korea.

From a clinical perspective, the classification moves beyond the general recommendation to “spend time in nature” by supporting the process of identifying and selecting park and green space resources and nature-based activities that are appropriate for individual patients' health conditions and life circumstances.

Primary care physicians and public health practitioners may first assess patients' health status, mobility, potential barriers to participation, and preferred activities, and then use the classification system to identify appropriate park characteristics and activity types. Park prescription guidance materials emphasize the importance of matching patients with appropriate parks by considering factors such as accessibility, activity options, safety, available programs, and proximity (53). Similarly, Park Prescription training materials guide the evaluation of health needs, barriers to participation, transportation options, community programs, and active living goals through patient case scenarios (54). In the present classification, for example, for an older adult with a chronic condition such as diabetes, healthcare providers may prioritize nearby parks that offer gently graded walking routes, and opportunities for rest, while recommending low-intensity aerobic activities such as walking combined with nature appreciation. For patients experiencing mild depressive symptoms or seeking stress reduction, parks that provide opportunities for landscape appreciation and nature experiences may be more appropriate. For patients with mobility limitations, the range of suitable parks may be narrowed based on considerations such as public transport or vehicle accessibility, facilities for vulnerable user groups, and safe conditions for use. More broadly, the classification may also support the design of Park Prescribing approaches that consider the characteristics and needs of different user groups, including children, adults, and older adults, in addition to patients with specific health conditions.

Similar uses of structured park information can also be found in existing Park Prescribing programs. Park Rx America provides a searchable park database that allows users to identify and compare parks based on a range of criteria, including accessibility, facilities, activities (55). This example suggests that such information can support the selection of park resources that correspond to patients' health goals and use conditions. Expert feedback further supported this potential application. Some experts proposed that a useful approach may be to identify activities that correspond to a patient's health goals before selecting specific parks, and then progressively narrow the available options using information on park and green space characteristics and spatial components. This activity-oriented decision-making approach may provide a useful pathway for applying Park Prescribing in clinical settings. Several experts also suggested that, to be useful in actual prescription settings, each item should be accompanied by guidance on how it relates to specific health needs or user characteristics.

From a policy perspective, the present classification may provide a basis for organizing Park and Green Space Service information and linking it with healthcare institutions in future Park Prescribing systems.

Local governments and park management agencies may use the classification to identify park resources relevant to Park Prescribing. Such information may serve as a foundation for establishing health-oriented approaches to park planning, design, and management. The resulting information may also be organized into a standardized database and provided to healthcare providers and users in the form of map-based park information or prescription-support materials. Furthermore, data on user needs and activity patterns accumulated through the prescription process may serve as a valuable resource for future park planning, management, and service improvement.

Given the expansion of preventive healthcare and chronic disease management policies led by the National Health Insurance Service in Korea, Park Prescribing may be considered a complementary health-promotion approach that can be integrated with existing preventive healthcare programs. As Park Prescribing becomes integrated with such programs, Park and Green Space Service information could also be linked with medical information systems, such as electronic health records (EHRs), to support the ongoing management of prescription processes and utilization outcomes. To support this process, collaboration among local governments, park management agencies, and healthcare institutions will be needed to incorporate Park and Green Space Service information into prescription support and utilization outcome management.

### 4.5 Limitations and future directions

The limitations of this study and directions for future research are as follows.

First, the overall expert panel consisted of 20 experts, meeting commonly recommended criteria for Delphi studies and providing an adequate basis for content validity assessment at the full-panel level. However, comparisons between the Healthcare group and the Park and Green Space group should be interpreted as exploratory because each group included only 10 experts. In addition, some provisionally retained items reflected the research team's overall judgement despite not fully meeting the predefined consensus criteria. Their appropriateness should therefore be re-examined through further Delphi surveys and implementation-based studies.

Second, although the expert panel included individuals with research or professional experience in the Healthcare and Park and Green Space fields, it may not have fully represented frontline practitioners directly involved in prescribing, coordinating, or supporting Park Prescribing, such as general practitioners and community nurses. In addition, because the classification was developed through expert consensus, the experiences, preferences, and priorities of patients and park users regarding prescribable park attributes were not directly incorporated. Future studies should therefore examine the applicability and interpretability of the classification with the participation of frontline practitioners, patients, and park users. In particular, qualitative research exploring how individuals with chronic conditions select and use park environments may provide valuable insights for complementing this expert-based classification framework.

Third, the practical applicability of the classification has not yet been validated in real-world prescribing contexts. In particular, no empirical evaluation has been conducted regarding patient adherence, healthcare provider usability, or implementation feasibility in clinical settings. Moreover, the classification comprises 101 Sub-Items and was not intended for all items to be used at the same level of detail in routine clinical practice. While the full classification may support park service assessment, planning, and management, clinical applications may require a more selective use of core items. Future research should therefore evaluate the applicability of the classification in actual Park Prescribing programs and explore the development of a simplified clinical version or a staged selection framework centered on high-priority items.

Fourth, this study focused on the development of the Park and Green Space Service Classification and did not establish evidence regarding which park characteristics or activities are most appropriate for specific health conditions or patient groups. Furthermore, it remains unknown whether items demonstrating higher content validity during the validation process are associated with more effective prescribing outcomes in practice. Future studies should therefore examine the effectiveness of Park Prescribing across different health conditions and identify appropriate prescribing approaches for specific patient needs.

Fifth, this study did not examine variations associated with regional contexts or urban land-use characteristics within Korea. Differences in park infrastructure, population density, and the degree of integration with healthcare systems may exist across metropolitan, small-city, and rural settings, as well as residential and commercial areas, potentially influencing the applicability of the Park and Green Space Service Classification. Future research should therefore compare the applicability and generalizability of the classification across diverse regional and spatial contexts.

## 5 Conclusion

This study developed a Park and Green Space Service Classification to support the practical application of Park Prescribing for chronic disease prevention and health promotion. A preliminary item structure was developed through a review of international Park Prescribing programs and Park Assessment and Observation Tools. Subsequently, two rounds of a modified Delphi survey were conducted with a panel of 20 experts, comprising 10 Healthcare experts and 10 Park and Green Space experts.

As a result, the final classification was refined into three Domains—Park and Green Space Characteristics, Park and Green Space Spatial Components, and Park and Green Space Activities—comprising 11 Major Items, 36 Middle-Level Items, and 101 Sub-Items. The findings identified [Accessibility] and [User Convenience], [Pedestrian Space] and [Rest and Interaction Space], and [Physical Health Activities] and [Passive Recreation Activities] as key items within Park Prescribing.

The significance of the classification lies in its conceptualization of parks and green spaces as prescribable service resources that can be selected and matched according to patients' health goals and life circumstances, and in its systematic organization of these resources. By doing so, the study provides a foundation for supporting the application of Park Prescribing within the healthcare and park and green space contexts of Korea.

However, further validation is needed to examine the applicability of the classification in real-world Park Prescribing settings. Future research should develop practical implementation strategies that reflect the perspectives of frontline practitioners involved in prescribing, referral, and adherence support, and empirically examine how Park and Green Space Services can be organized and applied for different health conditions and user groups. Such efforts may contribute to a more concrete understanding of the clinical applicability and policy potential of Park Prescribing.

## Data Availability

The original contributions presented in the study are included in the article/Supplementary material, further inquiries can be directed to the corresponding author.
