# Supplementary material for: Development of a park and green space service classification to support park prescribing in Korea
Source: Front Public Health. 2026 Jul 30;14:1857595. doi: 10.3389/fpubh.2026.1857595 (PMC13469281; doi:10.3389/fpubh.2026.1857595)
Supplement: Supplementary file 1 [file Data_Sheet_1.pdf]

Supplementary Table 1.
Item Extraction Matrix

Item	Park Prescribing Program						Park Assessment and Observation Tool				
	1	2	3	4	5	6	7	8	9	10	11
Park Location	○						○				
Park Type	○										
Park Area	○										
Park Entrance			○				○				
Public Transportation	○		○				○	○			○
Parking	○		○				○	○			○
Lighting	○						○	○			○
Safety and Security	○						○				○
Convenience Facilities	○	○	○				○				○
Pet Facilities	○	○	○				○				
Commercial Facilities	○	○									
Accessible Facilities	○		○								
Regular Programs	○										
Special Programs	○										
Green Space Type							○				
Green Coverage Ratio							○				
Trail Difficulty	○	○						○			
Trail Length	○						○				
Trail Slope	○							○			○
Trail Width	○							○			
Trail Type	○							○			
Trail Pavement Condition	○						○	○			○
Trail Surface Type	○						○	○			○
Rest Facilities Along Trails	○						○	○			
Trail Walking Time				○	○						
Available Trail Activities			○								
Shade Availability Along Trails	○										
Water Facilities	○		○				○				
Waterside Space	○	○	○				○				
Fitness Facilities	○		○				○				○
Sports Facilities	○		○				○				○
Playground Availability		○	○					○			
Playground Size	○										
Playground Shade	○						○				
Playground Surface	○										
Playground Users											
Open Space Availability			○					○			
Open Space Size	○						○				
Open Space Shade	○										
Cultural Facilities	○	○	○				○	○			○
Recreational Facilities	○	○	○								
Urban Agriculture Facilities	○			○		○					
Individual Physical Activities	○	○	○	○		○			○	○	
Group Physical Activities	○					○			○	○	
Passive Recreation Activities	○		○						○	○	
Recreational Activities	○	○	○						○	○	
Nature Experience Activities				○		○					
Nature Observation Activities					○	○					
Pet Activities	○	○			○						
Community Activities				○	○	○					

Sources

1. Park Rx America (https://parkrxamerica.org/)
2. Kids in Parks Track Trail (https://www.kidsinparks.com/track-rx)
3. Staying Healthy in Nature Everyday (https://www.ebparks.org/programs/hphp/shine)
4. NHS Forest (https://nhsforest.org/)
5. Nature Prescription (https://www.rspb.org.uk/helping-nature/what-we-do/nature-prescriptions)
6. Our Natural Health Service (https://www.nature.scot/professional-advice/contributing-healthier-scotland/our-natural-health-service)
7. Community Park Audit Tool (CPAT)
8. Prescription Trails Assessment Worksheet
9. System for Observing Physical Activity and Recreation in Natural Areas (SOPARNA)
10. System for Observing Play and Recreation in Communities (SOPARC)
11. Seoul Neighborhood Park Health Promotion Assessment Tool

Note. ○ indicates that the item was identified in the corresponding Park Prescribing Program or Park Assessment and Observation Tool.

Supplementary Table 2.
Preliminary Item Set for Park and Green Space Service

Domain	Major Item	Middle-Level Item	Sub-Item
Park and Green Space Characteristics	General Characteristics	Park Location	1) Urban / Residential Type<br>2) Mountain Type<br>3) Indoor Type
		Park Type	1) Urban Parks<br>2) Natural Parks<br>3) Recreational Trails
		Park Area	1) Less than 1,500 m²<br>2) 1,500 m² or larger (approximately three basketball courts)<br>3) 10,000 m² or larger (approximately 1.5 soccer fields)<br>4) 30,000 m² or larger (approximately four soccer fields)<br>5) 1,000,000 m² or larger
	Accessibility	Access Distance	1) Children's Living Area (within 250 m / 3-minute walk)<br>2) Neighborhood Living Area (within 500 m / 7-minute walk)<br>3) Walking Distance (within 1,000 m / 15-minute walk)<br>4) Regional Access (beyond 1,000 m / vehicle access)
		Public Transportation	1) Bus<br>2) Subway
		Parking Lots	1) Parking Lot<br>2) Bicycle Parking
	Safety	Lighting Facilities	1) On-site Lighting (excluding nearby streetlights)<br>2) No Lighting
		Management Facilities	1) Management Office<br>2) Safety Personnel<br>3) Emergency Call Devices and CCTV<br>4) Information on Park Maintenance and Cleanliness
	Usability	Service Facilities	1) Waste Bins, Restrooms, Drinking Fountains<br>2) Lockers, Umbrella Rental, Information Signage<br>3) Cafés, Restaurants, Vending Machines<br>4) Visitor Centers, Community Centers
		Pet Facilities	1) Dog Park (Off-Leash Dog Area)<br>2) Waste Bag Dispensers<br>3) Leash Policy (On-Leash / Off-Leash)
		Convenience	1) Barrier-Free Pathways

		Facilities	2) Wheelchair Rental<br>3) Facilities for Pregnant Women<br>4) Baby Care Room
	Program	Personal Program	1) Individual Physical Activities<br>2) Individual Relaxation Activities
		Community Program	1) Group Physical Activities<br>2) Community Participation Activities
	Naturalness	Natural Landscape	1) Green Landscape<br>2) Waterside Landscape<br>3) Scenic Viewpoints
		Green Space	1) Flower Beds, Wildflower Meadows<br>2) Lawns<br>3) Forests<br>4) Gardens
		Green Coverage Ratio	1) 0–25%<br>2) 25–50%<br>3) 50–75%<br>4) 75–100%
Park and Green Space Spatial Components	Pedestrian Space	Difficulty Level	1) Easy (Paths with paved, level surfaces and no obstacles)<br>2) Moderate (Paths with gentle slopes or minor surface variations)<br>3) Difficult (Paths with varying slopes)
		Pavement Type	1) Paved Trails<br>2) Unpaved Trails<br>3) Acupressure Walking Paths<br>4) Barefoot Walking Paths
		Length	1) Less than 1 km<br>2) 1–3 km<br>3) More than 3 km
		Width	1) Single-Person Walking (Effective width: 1.2–1.5 m)<br>2) Two-or-More-Person Walking (Effective width: ≥2.0 m)<br>3) Four-or-More-Person Walking
		Trail Layout	1) Loop Trail<br>2) Linear Trail<br>3) Not Applicable
		Activity Categories	1) Walking, Running<br>2) Cycling<br>3) Hiking

	Exercise Space	Fitness Facilities	1) Availability and Types of Outdoor Fitness Equipment
		Sports Facilities	1) Basketball Court<br>2) Volleyball Court<br>3) Baseball Field<br>4) Tennis Court<br>5) Futsal Court<br>6) Soccer Field<br>7) Gateball Court<br>8) Badminton Court<br>9) Swimming Pool
		Open Space	1) Lawn Area<br>2) Sports Field<br>3) Plaza
	Rest Space	Rest Facilities	1) Benches, Tables<br>2) Pavilions, Pergolas
		Play Facilities	1) Children's Playground<br>2) Nature Play Area
	Water Related Space	Water Features	1) Fountain<br>2) Pond, Lake<br>3) Waterfall, Cascade Wall<br>4) Stream<br>5) Water Playground
		Waterfront Space	1) River<br>2) Reservoir, Dam<br>3) Sea<br>4) Tidal Flats, Wetlands
	Cultural Space	Cultural Facilities	1) Library<br>2) Museum, Exhibition Hall<br>3) Outdoor Theater, Performance Venue
		Leisure Facilities	1) Campsite<br>2) Campground<br>3) Picnic Area<br>4) Arboretum, Botanical Garden
		Community Facilities	1) Garden Plots, Greenhous<br>2) Community Garden
Park and Green Space	Physical and Mental Health	Aerobic Exercise	1) Walking, Jogging, Running, Hiking<br>2) Cycling, Jump Rope, Inline Skating

Activities	Activities		3) Pet Activities<br>4) Aerobics, Dance Sports
		Resistance Exercise	1) Pull-Ups, Horizontal Bar Exercises<br>2) Outdoor Fitness Equipment Exercises<br>3) Climbing
		Flexibility Exercise	1) Stretching<br>2) Yoga
		Sports Activities	1) Ball Sports<br>2) Racket Sports
		Rest and Recreational Activities	1) Meditation, Napping<br>2) Conversation<br>3) Picnicking, Reading, Card Games
	Nature Based Activities	Landscape Appreciation Activities	1) Landscape Appreciation<br>2) Weather Appreciation
		Nature Observation Activities	1) Plant Observation<br>2) Wildlife Observation<br>3) Birdwatching
		Nature Learning Activities	1) Plant Learning<br>2) Park Exploration and Ecological Interpretation<br>3) Gardening and Horticultural Activities<br>4) Urban Agriculture Activities
	Community Program Activities	Cultural Activities	1) Attending Performances and Exhibitions<br>2) Participating in Festivals and Events
		Educational Activities	1) Recreational Sports Education<br>2) History and Environmental Education<br>3) Arts and Cultural Education
		Volunteer Activities	1) Park and Green Space Management Volunteering<br>2) Park and Green Space Program Volunteering

Supplementary Table 3.
Refinement of Middle-Level Items Across Delphi Rounds

Domain	Round 1			Round 2			Final Classification	
	**Major Item**	**Middle-Level Item**	**Changes**	**Major Item**	**Middle-Level Item**	**Changes**	**Major Item**	**Middle-Level Item**
Park and Green Space Characteristics	General Characteristics (3)	Park Location	[1]	General Characteristics (3)	1. Type	[1.1]	General Characteristics (3)	1.1 Type
		Park Type	[1]		2. Area	[1.2]		1.2. Area
		Park Area	[2]		3. Crowding Level †	[1.3]		1.3 User Density
	Accessibility (3)	Access Distance	[4]	Accessibility (4)	4. Pedestrian Accessibility	[2.1]	Accessibility (4)	2.1 Pedestrian Accessibility
		Public Transportation	[5]		5. Public Transportation Accessibility	[2.2]		2.2 Public Transportation Accessibility
		Parking Lots	[6]		6. Vehicle Accessibility	[2.3]		2.3 Vehicle Accessibility
					7. Cost Accessibility †	[2.4]		2.4 Cost Accessibility
	Safety (2)	Lighting Facilities	[10]	Safety (3)	8. Monitoring and Emergency Support	[3.1]	Safety (3)	3.1 Monitoring and Emergency Support
		Management Facilities	[8]		9. Risk Prevention †	[3.2]		3.2 Risk Prevention
					10. Safe Use Conditions	[3.3]		3.3 Safe Use Conditions
	Usability (3)	Service Facilities	[11]	User Convenience (3)	11. Amenities	[4.1]	User Convenience (3)	4.1 Amenities
		Pet Facilities	[11]		12. Vulnerable Group Facilities	[4.2]		4.2 Vulnerable Group Facilities
		Convenience Facilities	[12]		13. Program Offerings	[4.3]		4.3 Program Availability
	Program (2)	Personal Program	[13]					
		Community Program	[13]					
	Naturalness (3)	Natural Landscape	[14]	Natural Landscape Quality (3)	14. Green and Blue Space Type	[8.1]		
		Green Space	[14]		15. Landscape Characteristics †	[8.2]		
		Green Coverage Ratio	delete		16. Ecological Quality †	delete		
Park and Green Space Spatial Components	Pedestrian Space (6)	Difficulty Level	[17]	Pedestrian Space (3)	17. Difficulty Level	[5.1]	Pedestrian Space (3)	5.1 Difficulty Level
		Pavement Type	[19]		18. Length	[5.2]		5.2 Length
		Length	[18]		19. Activity Categories	[5.3]		5.3 Activity Type
		Width	delete					
		Trail Layout	delete					
		Activity Categories	[19]					
	Exercise Space (3)	Fitness Facilities	[20]	Exercise Space (3)	20. Fitness Facilities	[6.1]	Exercise Space (4)	6.1 Fitness Facilities
		Sports Facilities	[21]		21. Sports Facilities	[6.2]		6.2 Sports Facilities
		Open Space	[22], [24]		22. Open Exercise Areas	[6.3]		6.3 Open Exercise Areas
								6.4 Play

								Facilities
	Rest Space (2)	Rest Facilities	[23]	Rest and Interaction Space (3)	23. Rest Facilities	[7.1]	Rest and Interaction Space (4)	7.1 Rest Facilities
		Play Facilities	[26], [28]		24. Open Rest Areas	[7.2]		7.2 Open Rest Areas
					25. Cultural Facilities	delete		7.3 Leisure Facilities
								7.4 Gardening Facilities
	Water Related Space (2)	Water Features	[27], [14]	Play Space (3)	26. Age Specific Play Facilities	[6.4]		
		Waterfront Space	[14]		27. Seasonal Play Facilities	[6.4]		
					28. Nature Play Facilities	[6.4]		
	Cultural Space (3)	Cultural Facilities	[25]	Nature Experience Space (3)	29. Observation Facilities †	[8.3]	Nature Space (3)	8.1 Green and Blue Space Type
		Leisure Facilities	[30]		30. Leisure Facilities	[7.3]		8.2 Natural Landscape Type
		Community Facilities	[31]		31. Cultivation Facilities	[7.4]		8.3 Nature Experience Facilities
Park and Green Space Activities	Physical and Mental Health Activities (5)	Aerobic Exercise	[32]	Physical Health Activities (3)	32. Aerobic Exercise	[9.1]	Physical Health Activities (3)	9.1 Aerobic Exercise
		Resistance Exercise	[33]					9.2 Strength Training
		Flexibility Exercise	[34]					9.3 Sports and Group Exercise
		Sports Activities	[34]		33. Strength Training	[9.2]		
		Rest and Recreational Activities	[37]		34. Sports and Group Exercise	[9.3]		
	Nature Based Activities (3)	Landscape Appreciation Activities	[35]	Passive Recreation Activities (3)	35. Landscape Appreciation Activities	[10.1]	Passive Recreation Activities (3)	10.1 Landscape Appreciation Activities
		Nature Observation Activities	[36]		36.Nature Observation Activities	[10.2]		10.2 Nature Observation Activities
		Nature Learning Activities	[36], [39]		37. Rest and Recreational Activities	[10.3]		10.3 Rest and Recreational Activities
	Community Program Activities (3)	Cultural Activities	[38]	Community Program Activities (3)	38. Educational and Cultural Activities	[11.1]	Program Activities (3)	11.1 Physical and Cultural Activities
		Educational Activities	[38]		39. Nature Experience Activities	[11.2]		11.2 Nature Experience Activities
		Volunteer Activities	[40]		40. Volunteer Activities	[11.3]		11.3 Volunteer Activities

Note. Numbers in the Changes columns indicate modifications described in the item revision process. † indicates that the item was added in Round 2 based on the results of Round 1.

Supplementary Table 4.
Detailed Final Park and Green Space Service Classification

Domain	Major Item	Conceptual Definition	Middle-Level Item	Conceptual Definition	Sub-Item
Park and Green Space Characteristics	General Characteristics	Basic information describing the characteristics of parks and green spaces, including their type, area, and user density.	Type	Types of parks and green spaces based on geographic location and characteristics.	1) Neighborhood type<br>2) Neighborhood nature type<br>3) Remote nature type<br>4) Theme type
			Area	The size of a designated park or green space.	1) 1,500㎡(approximately 4 times the size of a basketball court, small green space) or less<br>2) 10,000㎡(approximately 1.5 times the size of a soccer field, small park) or less<br>3) 100,000㎡(approximately 15 times the size of a soccer field, neighborhood park) or less<br>4) 1 million㎡(approximately 150 times the size of a soccer field, wide-area neighborhood park) or less<br>5) Greater than 1 million㎡
			User Density	Perceived spatial characteristics based on the density of park and green space users.	1) Vital (Greater than 70% in density)<br>2) Normal (Greater than 50% ~ 70% or less in density)<br>3) Relaxing (Greater than 30% ~ 50% or less in density)<br>4) Quiet (30% or less in density)
	Accessibility	Physical and financial accessibility to parks and green spaces.	Pedestrian Accessibility	Walking distance and travel time from residential areas to the park.	1) Within a 5-minute walk (500m radius)<br>2) Within a 10-minute walk (1,000m radius)<br>3) Within a 15-minute walk (1,500m radius)<br>4) At least a 15-minute walk
			Public Transportation Accessibility	Information on public transportation providing access to the park entrance or nearby areas.	1) Bus<br>2) Subway<br>3) Other public transportation
			Vehicle Accessibility	Available parking spaces for accessing the park by car.	1) Parking available<br>2) No parking available
			Cost Accessibility	Information on whether use of the park or its parking facilities is free or requires fees, such as admission or parking charges.	1) Free use<br>2) Paid use<br>3) Free parking lot<br>4) Paid parking lot
	Safety	Safety facilities and management measures that support safe activities in	Monitoring and Emergency Support	Safety facilities that monitor crime and accidents or enable rapid emergency response.	1) Management and monitoring facilities<br>2) Emergency support facilities

		parks and green spaces through monitoring, emergency response, risk prevention, and safe use conditions.	Risk Prevention	Warning signs and safety facilities installed to prevent accidents in hazardous areas.	1) Warning and alert facilities<br>2) Risk prevention facilities
			Safe Use Conditions	Facilities that provide safe pedestrian movement free from vehicle interference and support safe activities at night.	1) Lighting facilities<br>2) Separate pedestrian paths
	User Convenience	Availability of facilities and programs that enable all users, including socially vulnerable groups, to access and use parks and green spaces conveniently.	Amenities	Facilities that support convenient use of the park.	1) Amenities<br>2) Retail facilities<br>3) Information facilities<br>4) Rental and storage facilities<br>5) Pet-friendly facilities<br>6) Facilities considerate of non-pet users
			Vulnerable Group Facilities	Facilities that enable socially vulnerable users to access parks and green spaces safely and conveniently, or to use facilities within the park.	1) Facilities for people with disabilities<br>2) Facilities for older adults<br>3) Facilities for pregnant women and infants
			Program Availability	Programs operated by park management or related organizations that allow individuals or groups to experience the park in diverse ways.	1) Individual programs<br>2) Group programs
Park and Green Space Spatial Components	Pedestrian Space	Spaces used for walking, strolling, movement, and pedestrian circulation within parks and green spaces.	Difficulty Level	Walking difficulty based on slope and surface conditions.	1) Easy<br>2) Moderate<br>3) Difficult
			Length	Round-trip distance from the trail entrance to the viewpoint, or the total distance of a loop trail.	1) 1km or less<br>2) 1-3km<br>3) Greater than 3km
			Activity Type	Activity characteristics or types supported by the surface type and condition of the walking space.	1) Walking, running<br>2) Cycling<br>3) Hiking<br>4) Accessible trails
	Exercise Space	Spaces equipped with facilities or open areas that support exercise and physical activities for fitness	Fitness Facilities	Exercise facilities that support basic physical fitness through aerobic exercise, strength training, stretching, and related movements.	1) Outdoor exercise equipment<br>2) Gymnastics equipment<br>3) Climbing facilities<br>4) Tracks

		improvement or recreational purposes.	Sports Facilities	Sports facilities designed for individual or group sports activities.	1) Ball-sport facilities<br>2) Racket-sport facilities
			Open Exercise Areas	Open spaces where users can exercise and engage in activities freely without exercise equipment.	1) Sports field<br>2) Plaza
			Play Facilities	Facilities that promote physical movement and enjoyment through play activities.	1) Age specific play facilities<br>2) Seasonal play facilities<br>3) Nature play facilities
	Rest and Interaction Space	Spaces where users can experience nature, rest physically and mentally, interact with the natural environment, and engage in social interaction within parks and green spaces.	Rest Facilities	Facilities for sitting or lying down for rest or contemplation.	1) Benches<br>2) Pavilions<br>3) Shelters
			Open Rest Areas	Open spaces where users can rest with family or friends.	1) Lawn area<br>2) Waterside rest area
			Leisure Facilities	Spaces where users can spend time in nature and rest.	1) Natural recreational facilities<br>2) Camping facilities
			Gardening Facilities	Facilities where individuals or communities can cultivate crops, trees, or flowers while interacting and resting.	1) Individual garden plots<br>2) Community garden plots<br>3) Community gardens
	Nature Space	Spaces that include green and blue space elements, scenic landscapes, and facilities for nature-based experiences in parks and green spaces.	Green and Blue Space Type	Types of natural or designed spaces where users can experience nature, rest, and enhance emotional well-being while viewing green spaces or water features.	1) Natural spaces<br>2) Designed and constructed spaces
			Natural Landscape Type	Types of landscapes with visual quality and diversity that can be appreciated and enjoyed.	1) Open landscapes<br>2) Enclosed landscapes
			Nature Experience Facilities	Facilities that support nature-based exploration, including walking through natural landscapes and closely observing diverse ecosystems, plants, and animals.	1) Landscape appreciation facilities<br>2) Ecological observation facilities
Park and Green Space Activities	Physical Health Activities	Activities focused on promoting physical activity through individual or	Aerobic Exercise	Exercises such as walking, running, and cycling that increase oxygen consumption and improve heart and lung function.	1) Low-intensity (1.5 MET or more - less than 3.0 MET)<br>2) Moderate-intensity (3.0 MET or more - less than 6.0 MET)<br>3) High-intensity (6.0 MET or more)

		group exercise performed in parks and green spaces while taking advantage of outdoor environments.	Strength Training	Exercises that improve skeletal muscle strength, endurance, power, and muscle mass.	1) Equipment exercises<br>2) Bodyweight exercises
			Sports and Group Exercise	Competitive activities performed by groups according to established rules, or group-based exercise activities.	1) Sports activities<br>2) Group exercises
	Passive Recreation Activities	Activities focused on emotional restoration and mental well-being through appreciating and experiencing the beauty and diversity of nature while resting in natural environments.	Landscape Appreciation Activities	Activities involving rest while appreciating natural or local landscapes and their characteristics.	1) Landscape appreciation<br>2) Weather appreciation
			Nature Observation Activities	Activities involving close observation and sensory experience of plants and animals with interest in natural ecosystems.	1) Plant observation<br>2) Wildlife observation
			Rest and Recreational Activities	Activities that provide physical and mental rest in nature and enjoyment through social interaction.	1) Meditation and contemplation<br>2) Recreational activities<br>3) Social activities
	Program Activities	Activities focused on leisure and social interaction through participation in sports, cultural, experiential, and volunteer programs offered at parks and green space facilities.	Physical and Cultural Activities	Programs offered at park facilities that support leisure and social interaction through diverse recreational, physical, and cultural activities.	1) Group exercise programs<br>2) Arts and cultural activities
			Nature Experience Activities	Nature-based experiences and learning activities that support connection with nature and emotional well-being.	1) Nature exploration programs<br>2) Gardening and therapeutic farming activities<br>3) Ecological play programs
			Volunteer Activities	Volunteering activities that help maintain the park environment or support programs operated in parks and green spaces.	1) Park and green space maintenance volunteering<br>2) Park and green space program operation volunteering

Note. Shaded cells indicate provisionally retained major or middle-level items.

Supplementary Table 5.
Expert Group-Specific Analysis of Non-Consensus Middle-Level Items

Domain	Major Item	Middle-Level Item	Area of Expertise	Mean	S.D.	CVR	Convergence	Consensus	Stability
Park and Green Space Characteristics	General Characteristics	Area	Healthcare	3.800	.400	.600	.000	1.000	.105
			Park and Green Space	3.700	.640	.200	.500	.750	.173
	Natural Landscape Quality	Ecological Quality	Healthcare	3.400	.663	.000	.500	.714	.195
			Park and Green Space	4.200	.600	.800	.375	.813	.143
Park and Green Space Spatial Components	Exercise Space	Fitness Facilities	Healthcare	4.200	.600	.800	.375	.813	.143
			Park and Green Space	4.000	.894	.200	1.000	.500	.224
		Sports Facilities	Healthcare	3.800	.748	.200	.500	.750	.197
			Park and Green Space	3.900	.700	.400	.375	.813	.179
	Rest and Interaction Space	Cultural Facilities	Healthcare	3.600	.490	.200	.500	.750	.136
			Park and Green Space	3.900	.700	.400	.375	.813	.179
	Play Space	Age Specific Play Facilities	Healthcare	3.700	.458	.400	.375	.813	.124
			Park and Green Space	4.000	.775	.400	.750	.625	.194
		Seasonal Play Facilities	Healthcare	2.700	.640	-1.000	.000	1.000	.237
			Park and Green Space	3.900	.539	.600	.000	1.000	.138
		Nature Play Facilities	Healthcare	3.400	.490	-.200	.500	.667	.144
			Park and Green Space	4.300	.640	.800	.500	.750	.149
	Nature Experience Space	Observation Facilities	Healthcare	3.400	.490	-.200	.500	.667	.144
			Park and Green Space	4.000	.775	.400	.750	.625	.194
		Leisure Facilities	Healthcare	3.500	.500	.000	.500	.714	.143
			Park and Green Space	4.000	.447	.800	.000	1.000	.112
		Cultivation Facilities	Healthcare	3.400	.490	-.200	.500	.667	.144
			Park and Green Space	3.900	.700	.400	.375	.813	.179
Park and Green Space Activities	Passive Recreation Activities	Nature Observation Activities	Healthcare	3.600	.490	.200	.500	.750	.136
			Park and Green Space	4.300	.781	.600	.500	.778	.182
	Community Program Activities	Educational and Cultural Activities	Healthcare	3.600	.490	.200	.500	.750	.136
			Park and Green Space	3.700	.781	.400	.375	.813	.211
		Nature Experience Activities	Healthcare	3.600	.490	.200	.500	.750	.136
			Park and Green Space	4.000	.775	.400	.750	.625	.194
		Volunteer Activities	Healthcare	3.600	.663	.000	.500	.714	.184
			Park and Green Space	3.800	.748	.200	.500	.750	.197

Note. This table presents expert group-specific results for middle-level items that did not meet the predefined Round 2 Delphi criteria. CVR was interpreted using a threshold of ≥ .62 for each expert group. Shaded cells indicate items that met the expert group-specific criteria: CVR ≥ .62, convergence ≤ .50, consensus ≥ .75, and stability ≤ .50.
